# Round the Hospitals

**Published:** 1918-12-14

**Authors:** 


### ROUND THE HOSPITALS.

### The Queen to the Women of the Empire.

Buckingham Palace.

**A few months ago, at the height of our anxiety and strain, I sent a message in the name of the women of our lands to our men fighting for us across the seas. Now, in an hour of thankfulness and hope, I should like to give a message to the women of the Empire.**

**During the war they have been given the high privilege of service; they have risen to the great opportunity and have proved their courage, steadfastness, and ability. I have been allowed to watch and appreciate their work in many parts of the country, and my heart is full of admiration and gratitude for what I have seen.**

**I earnestly trust that though the thrill and glamour of war are over, the spirit of self-sacrifice and helpfulness which it has kindled will not wane in the coming days. A new era is dawning upon the world, bringing with it many difficulties, fresh responsibilities, and serious problems to be faced.**

**Parliament has secured for the whole country greater opportunities of more thorough and varied education, but it will depend upon the parents whether these opportunities are used to the full.**

**We all rejoice that plans are afoot for bringing to an end the existence of such bad and crowded housing as makes home life almost impossible.**

**To-day, more than ever, the Empire needs her daughters. For in the larger world of public and industrial work women are daily taking a more important place.**

**As we have been united in all our work, whether of head or hands, in a real sisterhood of suffering and service during the war, let us go on working together with the same unity of purpose for the resettlement and reconstruction of our country.**

**MARY R.**

The College of Nursing has followed Her Majesty's lead and given those nurses who are members of it a list of questions which they are urged to put to candidates in the Parliamentary Election in support of the College Bill (see p. vi) God helps those who help themselves, and the lead of the Council in this matter may well enthuse every member of the College to action, in their own and the College's progress. It is time the members began to recruit 10,000 additional nurses for the College Register. Every certificated nurse who loves her profession and has self-respect, should join the College by the end of February, before the £500,000 now being raised for the Nurses' Tribute is paid to the bankers. The organisation is in able hands, and the money is bound to flow in steadily and quickly now the means are forthcoming to raise it. Let every trained nurse awake right away and work for the College and her own best interests.

The inspiring words of the Queen to the women of the Empire ought not merely to be glanced through in the newspapers as an item of the morning's news. They ought to be read aloud in every ward, in every class-room, where women are preparing for their life-work as healers, teachers, and reconstructors generally. The Queen has shown herself a fitting leader of modern women. She did much, and very much, to bring women of all classes during the war to realise their unity and common membership of "a real sisterhood of suffering and service." She is already stimulating the spirit of self-sacrifice and helpfulness which she warns us all will need in the new era dawning upon the world. Where she leads, women will gladly and hopefully follow. Her message, then, is to be handed on by women in authority to all classes in the home or in the institution. We would have the whole household gathered together so far as is possible, not forgetting the maids whose part has been so honourable through years of under-staffing, and the message read slowly and distinctly with a touch of ceremony so that each person present may realise it is addressed to her personally.

There is one aspect of women's part in the war which is ever present to observant eyes, and which causes increasing disquiet. It is the spirit of recklessness among the younger workers. Partly, no doubt, it is due to the overwhelming number of soldiers in the streets eager for amusement. But this does not account entirely for the boisterous behaviour, the unrestrained impulses for excitement of any kind shown by many thousands of the younger women in the present day. Their up-bringing is quite obviously deficient in many directions. Physically, mentally, and morally they are completely unbalanced. The Queen glances at the difficulty when she alludes to the responsibility of parents in making use for their children of the more thorough and varied education in view. The country wants a lead in the solution of the difficult problems connected with the girl hooligan. She has plenty of good impulses and much undeveloped talent all running to waste. She is imperatively needed to take part in reconstruc-

**ROUND THE HOSPITALS—(*continued*).**

tion. But she is a waste product fitted only for the roughest manual labour, terribly, terribly unfitted for motherhood.

THAT well-tried maxim in nursing reforms, "What is the good of it?" echoes ominously among women voters as the election draws near. To all doubters and hesitators, to the sluggish, the uninterested, the petty-minded (if any such can be found in the nursing world), we commend Mr. Lloyd George's stirring admonition that it is better for women to vote amiss than not to vote at all. When the votes are counted it must not be said that British women cared so little about the larger issues of national life that only an inconsiderable number of them took the trouble to go to the poll. On women in positions of authority there rests the obligation not merely of recording their own vote, but also of driving—we use the word advisedly—other women to record theirs. It is one of those cases where a little zeal can leaven the whole lump.

THE Health Committee at Birmingham have taken strong measures in their fight with the influenza epidemic. The great need is for nurses, and this is being met as far as possible by an admirable system of organisation in the Birmingham District Nursing Association, by means of which nearly 9,000 visits were paid in November by the staff. Health visitors, emergency nurses, and V.A.D. workers are all helping. In many of the poorer families the bed-clothes are pitifully inadequate, and the public is asked for gifts of blankets, bed-linen, and towels. The hospitals are working at high pressure and with, in most cases, a sadly depleted staff.

VERY touching and impressive was the meeting of the old friends and fellow-workers of Miss Ray, R.R.C., at King's College, on December 5, when a cheque for £254 was presented to her, with an album beautifully illuminated with the arms of the hospital and an inscription, the work of one of the nurses. It contained the autographs of some 300 signatories, of whom a good proportion managed to be present. Lady Hambleden, who had the honour of making the presentation, spoke with much feeling of the love and admiration felt by all who had been associated with Miss Ray during her many years' work at King's College Hospital. A magnetic personality in the highest sense, Miss Ray has been the centre of a strong and uplifting influence throughout her whole term of office, a force making for equilibrium, steadying all who came under her sway to a deeper sense of their own powers, and a more perfect realisation of the uses of life. Many were visibly moved during the little ceremony, but there is much consolation in the knowledge that Miss Ray will from now be set free to throw the great charm of her influence in nursing matters into the weightiest scale.

THE system of training nurses at the Western Infirmary, Glasgow, is very thorough and complete, owing in no small measure to the hearty co-operation of members of the medical profession. That this fact is recognised is shown by the number of applications for admission to training, viz. 469, though the number of vacancies to be filled was only sixty-three. The managers express their pleasure, and their hearty congratulations to the matron, Miss Gregory Smith, on her appointment as a member of Queen Alexandra's Army Nursing Board. It is interesting to note how the fashion of the moment is involving the voluntary hospitals, however successful. The Western Infirmary authorities have, for instance, increased the pay granted to probationers to £17—that is, from £10 to £17 in the first year, and by £7 a year during each of the three remaining years on the salaries previously paid. Staff nurses now receive £40, plus a bonus of £5, and ward sisters £50, plus a bonus of £10.

THE annual prize-giving on December 3 at the Bristol General Hospital excited unusual interest, and brought together a very large gathering of nurses and others. Mr. G. A. Wills presided. The Chairman of Committee, Mr. Herbert Baker, took occasion to express the debt of gratitude felt by the whole institution to Miss Densham, the matron, and to Dr. Albert Lim, for their untiring exertions throughout the recent epidemic of influenza, when between fifty and sixty of the staff were down with it. He conveyed also the thanks of the committee to the nursing staff and to Miss Cameron. The prizes awarded were as follows: Gold medal, Nurse Ethel Clark; silver medal, Nurse Eleanor Keene. Certificates of efficiency: Nurse Dorothy Morris, Nurse Beatrice Godfrey, Nurse Ethel Panes. The Lottie Culverwell Memorial Prize was given by Mrs. Samuel Hosegood to the best nurse of her year, Nurse Ethel Clark. First prize on an examination on medical nursing, Nurse Clarie Wood; second prize, Nurse Ethel Panes. First prize, surgical nursing, Nurse Ethel Panes; second prize, Nurse Irene North. First prize, physiology, Nurse Eleanor Keene; second prizes, Nurse Winifred Long and Nurse Dorothy Crocker. First prize, anatomy, Nurse Kate Wells; second prize, Nurse Nellie Moody. First prize, practical nursing, Nurse Jessie Franklin; second prize, Nurse Eva Heard. Great satisfaction was expressed at the starting of the local centre of the College of Nursing at Bristol during the year, an enterprise which has already become an assured success.

OUR readers may like to learn that the portrait of Princess Mary, who has a title to be reckoned *par excellence* the Nurses' Princess, is to be had from the Rev. Vernon Jones, St. John of Beverley's Institute, Green Lanes, Finsbury Park, N., price 10s. 6d. It is a second edition of Miss Worsford's portrait of the Princess in her V.A.D. dress, and is being sold for the benefit of the Deaf and Dumb Girls' Club of North London.

**ROUND THE HOSPITALS**—(*continued*).

Dr. Truby King gave the first of his two lectures under the National Health Society at 53 Berners Street, W. 1, on December 5, with Dr. Mary Scharlieb, C.B.E., in the chair. The lecturer, enforcing the truth that all that is best in us is derived from family life, exposed the need for many more educated women to become teachers in the homes as health visitors, sanitary inspectors, and superintendents of infant welfare centres, the demand being in excess of the supply.

The League of Nations Fair in aid of the Royal Free Hospital was opened on December 4 by Princess Marie Louise, who gave a little account of the valuable work done by the hospital and her own connection with it. The Princess visited all the stalls and bought some useful supplies—sugar, pickles, vegetables, etc.—for her hospital in Bermondsey. All the stalls did well, even the one for cakes and chocolates was well filled in spite of the Food Controller, and made over £100, its supplies being mainly gifts from the big hotels. Up to the time of going to press £2,300 has been realised, and more is expected, so that the total promises to exceed even the high expectations formed by that able organiser and friend of nurses, Lady Cowdray.

Thrilling adventures are reported by the nurses who were the first to penetrate through the Dardanelles to Constantinople after the signing of the Armistice. A letter from one of them has been published by the *Daily Sketch*, recounting the narrow escape of the hospital ships from being blown up in a mine-field. They landed at Sedd-el-Bahr, and were the first women to set foot on that tragic landing. They saw the Allied Fleet steam past, and following on ran out of their course the next day, and saw mines drifting within a few yards of them. They had a grand reception at Constantinople, where the city is rejoicing at the cessation of hostilities, and the end of Turkish rule. Even the large German population declares war was worth while if it has got rid of the Hohenzollerns. Crowds turned out to see the English nurses.

The nurses' duties among our prisoners at Constantinople exposed a pitiful depth of suffering and ill-treatment. "Many have been interned three years, and have endured terrible cruelty and almost incredible brutality at the hands of the Turks." Only 260 out of the hapless band of 3,000 survived the march of from 500 to 600 miles across the desert. "They were flogged and starved, and some had nothing on at all, and many were barefooted. Those who fell out were bayoneted by the Turks. It makes one's heart ache to hear much that they have to tell us." The nurses felt it a great honour to be chosen to minister to these men and bring them home.

To glance through the November number of the nursing journals from the other side of the Atlantic is to realise how great a gulf the events of November 11 have dug between the present and the past. Preoccupations about training war nurses in a hurry, yet guarding the nursing standard from being lowered; urgent appeals for more volunteers for Red Cross work, side by side with really pitiful accounts of the shortage of nurses for civil needs—these are the weighty matters which oppressed our Transatlantic sisters until the Armistice swept all such perturbations away. That things will never be quite the same in American nursing administration as they were prior to the past strenuous year is more than probable. As in Great Britain, so in the United States much has been learned, and some cherished doctrines may need re-stating.

Unless precautions are speedily taken by the Local Government Board the public will be exposed to grave dangers through the blunders committed in the founding and maintenance of a large number of homes for babies and mothers by persons totally unacquainted with the precautions necessary to be observed in establishments of this nature. There is an imperative call for the issue of regulations to safeguard the poorer classes in the present haphazard multiplication of small nursing homes by District Councils. We speak from knowledge when we assert that total ignorance as to the number and qualifications of the nursing staff requisite for even a small lying-in home or babies' centre is prevalent among members of committees appointed to organise these welfare schemes. They need authoritative advice in staffing their little institutions, and cannot safely be left to their own discretion. The appointment of one or more organising matrons to advise local committees on these matters and inspect ought not to be delayed.

